# Protic Ionic Liquids as Eco-Friendly Surfactants for Oil Spill Remediation: Synthesis, Characterization, and Performance Evaluation

**DOI:** 10.1021/acsomega.5c02170

**Published:** 2025-06-16

**Authors:** Sádwa F. Ribeiro, Hiago S. Braga, Rilvia S. Santiago-Aguiar

**Affiliations:** Chemical Engineering Department, Federal University of Ceara, Pici Campus, Bloco 709, Fortaleza, Ceará 60440-900, Brazil

## Abstract

This study presents an innovative approach by synthesizing and evaluating protic ionic liquids (PILs) as sustainable surfactants for oil spill remediation, as an alternative to the highly toxic chemical dispersants conventionally used, as well as to the surface-active ionic liquids reported in the literature, which are typically based on imidazolium cations and halogenated anions. Four PILs—butyrolactam butyrate (BTB), 2-methyl-hydroxyethylammonium propanoate (2-HEAP), 2-methyl-hydroxyethylammonium acetate (2-HEAA), and choline laurate [CHL]­[LAU]—were synthesized and analyzed for physicochemical and surfactant properties. Characterization included Fourier transform infrared (FTIR) and nuclear magnetic resonance (NMR) spectroscopy, while density, viscosity, surface tension, and emulsification performance were also evaluated. Among them, [CHL]­[LAU] exhibited the best performance, significantly reducing surface tension (22.7 mN/m) and forming stable emulsions with motor oil and kerosene. It also demonstrated an effective critical micellar concentration (CMC) of 29.9 mmol/L, enabling efficient hydrocarbon solubilization. In oil removal tests, [CHL]­[LAU] performed comparably to the commercial surfactant SDS, while in oil displacement area tests, it outperformed SDS, covering up to 93.5% of the surface. Toxicity tests using *Artemia salina* confirmed that [CHL]­[LAU] is less toxic than conventional surfactants. These findings highlight [CHL]­[LAU] as a promising and environmentally friendly surfactant for oil spill remediation, offering an effective and sustainable alternative to conventional dispersants.

## Graphical abstract

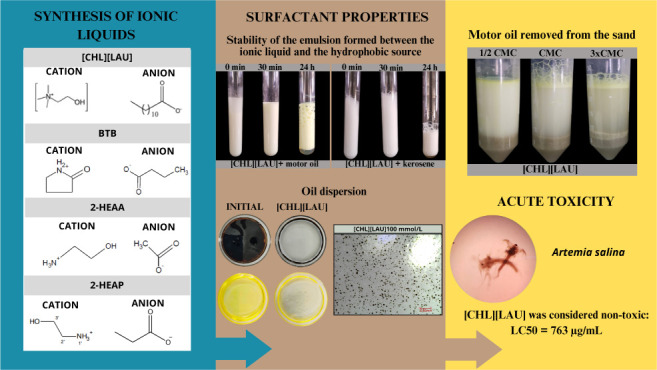

## 1 Introduction

The growing global demand for oil, accompanied by the rapid expansion of exploration, extraction and operations of the oil industry, has increased concerns about environmental issues, especially those related to the impacts resulting from oil spills at sea caused by structural failures in pipelines and oil platforms, collisions between oil tankers or shipwrecks, and operational errors during drilling activities.^1–3^ In this context, several medium and large scale spills have been recorded worldwide, including the Torrey Canyon in 1967 with 119000 tons of oil spilled, Amoco Cadiz in 1978 with 227000 tons, Exxon Valdez in 1989 with 37000 tons, Sea Empress in 1996 with 72000 tons, and the Deepwater Horizon, in the Gulf of Mexico in 2010, which released 4.9 million barrels of crude oil into the sea.^4,5^ In Brazil, the 2019 spill impacted over 1000 locations along 3000 km of coastline, affecting intertidal rocky shores, rhodolite beds, sandy beaches, mangroves, estuarine systems, seagrass beds, and coral reefs.^6^

Oil contamination can persist in the environment for many years after a spill, as can the effects resulting from these leaks.^7–9^ The high toxicity and complexity of the hydrocarbons found in crude oil, in addition to causing physiological damage to marine life, can contaminate the entire food chain, including marine mammals, birds and even humans.^10,11^ In addition, pollution caused by oil has a major economic impact, since cleanup costs are quite high and tourism and fishing activities are interrupted.^12,13^

To mitigate the impacts of such disasters, remediation techniques are essential. Chemical surfactants are widely used due to their ability to disperse oil into small droplets, facilitating biodegradation.^14^ This is possible thanks to their amphiphilic chemical structure that allows adsorption on surfaces and interfaces, in addition to their ability to aggregate in solutions to form micelles.^15^ However, most conventional surfactants are derived from petroleum, exhibiting high toxicity and low biodegradability.^16^ In the explosion of the Deepwater Horizon drilling platform, for example, approximately 6.81 million liters of chemical dispersants were applied to the water surface,^17^ including CorexitEC9500A, which has a lethal concentration (LC_50_) of 20.7 mg·L^–1^, characterizing it as a highly toxic substance.^18,19^

This highlights the need to develop more sustainable dispersants with improved physicochemical properties.^20,21^ In this context, the use of surfactants based on ionic liquids (ILs) for oil spill remediation presents itself as a promising alternative due to their tunable properties, including low volatility, thermal and chemical stability, high ionic conductivity and amphiphilicity.^22,23^ Several studies have explored the influence of surface-active ionic liquids (SAILs) on the surface properties of systems. This is important because one of the principles of dispersion of oil slicks into smaller droplets is the reduction of interfacial and surface tensions.

Jin et al.^24^ investigated the aggregation of 1-butyl-3-methylimidazolium dodecylsulfonate in aqueous solution and observed more than 33% reduction in surface tension, while Singh et al.^25^ studied the micellization behavior of imidazolium-based amphiphilic ionic liquids and found that the critical micelle concentration (cmc) obtained was 2 to 3 times lower compared to conventional surfactants. Nabipour et al.^26^ applied 1-dodecyl-3-methylimidazolium chloride ([C12mim]­[Cl]) and 1-octadecyl-3-methylimidazolium chloride ([C18mim]­[Cl]) for oil recovery by reducing the interfacial tension of the solution from 19 to 0.07 mN·m^–1^ and modifying the wettability. Shalfiee et al.^23^ studied the surface activities of asymmetric dicationic ionic liquids and obtained oil dispersion efficiencies ranging from 51.22% to 75.50% at a temperature of 323.15 K.

Although the aforementioned studies have obtained satisfactory results regarding the efficiency of surfactant ionic liquids, it is observed that they focus on substances synthesized from cations derived from imidazolium, pyridinium, pyrrolidinium, alkylammonium and morpholinium, in combination with halogenated anions, such as chlorine and fluorine.^21,27,28^ This composition generally results in compounds with high toxicity and low biodegradability, which poses significant risks to the environment. Given this scenario, it is imperative to search for more sustainable alternatives that combine efficiency in oil dispersion with lower environmental impact. In this context, the present work proposes the investigation of protic ionic liquids (PILs) as dispersing agents. Unlike aprotic ionic liquids—whose synthesis involves multiple steps, high cost and complex molecular structures, in addition to presenting high toxicity and limited biodegradability^21,29,30^ —PILs can be obtained through a simple neutralization reaction, involving the transfer of protons between an acid and a Brønsted base. In addition to ease of synthesis and lower cost, PILs are more environmentally compatible due to the less toxic nature of their precursors.^31^

Thus, the present study aims to synthesize and characterize four PILs: butyrolactam butyrate (BTB), 2-hydroxyethylammonium acetate (2-HEAA), 2-hydroxyethylammonium propanoate (2-HEAP), and choline laurate [CHL]­[LAU], an ionic liquid based on choline and fatty acid. Their surfactant activity was evaluated through surface tension and emulsification index measurements. The most effective PIL was then undergo acute toxicity testing with *Artemia salina* and be assessed for its oil spill remediation performance, focusing on hydrocarbon removal from sand and oil displacement efficiency.

## 2 Materials and Methods

### 2.1 Materials

Choline chloride (99%), γ-butyrolactam (99%), and sodium dodecyl sulfate (SDS) (99%) were purchased from Sigma-Aldrich Chemical Co. Butyric acid (99%) and acetic acid (99%) were obtained from Vetec Química Fina Ltd. Monoethanolamine (99%), potassium hydroxide (85%) and potassium dichromate (99%), sodium chloride (99%), and ethyl alcohol (95%) were obtained from Neon Comercial Reagentes Analíticos Ltd. Propanoic (99.5%) and lauric (98%) acids were obtained from Dinâmica Química Contemporânea Ltd. and Êxodo Cientifica Ltd., respectively. White quartz sand (50–70 mesh) was obtained from Sigma-Aldrich Chemical Co., TRM 5 engine oil and *Artemia salina* cysts were obtained from local shops in the city of Fortaleza, Ceara. The medium crude oil (24 °API), whose properties were presented in Table S2, was supplied by the Phase Equilibrium Laboratory of the Department of Chemical Engineering of the Federal University of Ceara.

### 2.2 Synthesis of Protic Ionic Liquids

The synthesis of butyrolactam butyrate (BTB) was performed according to the methodology described by Bessa et al.^32^ and involved an equimolar neutralization reaction between the cyclic amide γ-butyrolactam and butyric acid. The reaction was maintained under constant stirring at 300 rpm for 24 h in a container with cold water (293.15 K) to remove the heat generated during the neutralization. The resulting product was subjected to rotary evaporation at 338.15 K for 24 h. The same procedure^23^ was applied using acetic acid and ethanolamine to synthesize 2-methyl-hydroxyethylammonium acetate (2-HEAA), and pentanoic acid and ethanolamine to synthesize 2-methyl-hydroxyethylammonium propanoate (2-HEAP).

Choline hydroxide was prepared following a modified method from Fan et al.^33^ Equimolar amounts of choline chloride and potassium hydroxide were separately dissolved in ethanol under constant stirring and heating (323.15 K). The solutions were then combined in a glass reactor equipped with a stirrer and thermostatic bath, and the reaction proceeded at 333.15 K and 600 rpm for 24 h. After cooling to room temperature, potassium chloride (KCl) was removed via vacuum filtration using a Millipore filter with a polytetrafluoroethylene (PTFE) membrane (0.45 μm porosity). Finally, the choline hydroxide solution was rotary evaporated at 338.15 K under 20 kPa for 24 h to remove ethanol.

The synthesis of choline laurate was adapted from the method established by Shan et al.^34^ Lauric acid, previously dissolved in ethanol, was slowly added dropwise to choline hydroxide under continuous stirring (300 rpm) at room temperature (298.15 K) in a 1:1 molar ratio. The reaction was maintained for 24 h, and the final product was subjected to rotary evaporation at 338.15 K under 20 kPa for 24 h.

Table 1 shows the names, abbreviations, and chemical structures of the synthesized ionic liquids.

**Table 1:**
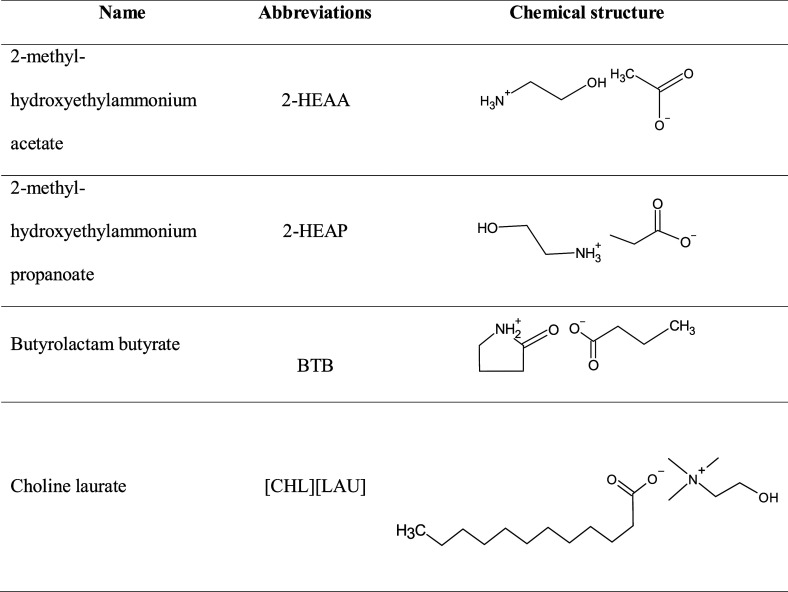
Names, Abbreviations, and Chemical Structure of the Ionic Liquids Produced

### 2.3 FTIR Analysis

FTIR analyses were conducted using a Cary 630 Fourier Transform Infrared Spectrometer (Agilent Technologies). Transmittance spectra were recorded in the range of 650–4000 cm^–1^ with a spectral resolution of 1 cm^–1^ and 32 scans.

### 2.4 ^1^H NMR of Synthesized Ionic Liquids

Proton nuclear magnetic resonance (^1^H NMR) spectroscopy was performed for the synthesized ionic liquids and their equimolar physical mixtures. Spectra were acquired using an Agilent DD2 600 MHz spectrometer equipped with a One Probe (5 mm internal diameter, HF/^15^N-^31^P) with inverse detection and a field gradient along the *z*-axis.

For sample preparation, 15 mg of each ionic liquid or physical mixture was dissolved in 550 μL of a specific deuterated solvent. Choline laurate ([CHL]­[LAU]) was analyzed in deuterated chloroform (CDCl_3_), while 2-hydroxyethylammonium acetate (2-HEAA), 2-hydroxyethylammonium propanoate (2-HEAP), and butyrolactam butyrate (BTB) were dissolved in deuterated dimethyl sulfoxide (DMSO-*d*_6_).

The ^1^H NMR experiment was conducted with a 2 s relaxation delay, an acquisition time (AQ) of 3.3 s, a gain of 26, and 32 transients within a 16 ppm spectral window, using 32k real points at 299.15 K. Spectra were referenced using tetramethylsilyl propionate (TMSP-*d*_4_) at 0 ppm, and chemical shifts were analyzed with MestReNova v6.0.2–5475 software.

### 2.5 Density and Viscosity

The density and viscosity of the synthesized ionic liquids were measured using an Anton Paar SVM 3000 digital viscometer with U-Tube oscillation, at atmospheric pressure (101.325 kPa) and within a temperature range of 298.15 to 333.15 K. Measurement uncertainties were 0.35% for viscosity and 0.0005 g/cm^3^ for density.

### 2.6 Emulsification Index and Emulsion Stability

Emulsions were prepared following the methodology described by Cooper and Goldenberg,^86^ with modifications. Briefly, 2 mL of the hydrocarbon source (kerosene or motor oil) was mixed with 2 mL of the ionic liquids BTB, 2-HEAA, 2-HEAP, and [CHL]­[LAU]. A 100 mM sodium dodecyl sulfate (SDS) solution served as the positive control, while deionized water was used as the negative control.

The mixtures were vigorously vortexed using a Q220 (QUIMIS) for 2 min, then left to stand at 298.15 K. Observations were recorded at 30 min and 24 h to assess stability. The emulsification index was determined after 24 h by calculating the ratio between the height of the emulsified layer and the total height of the mixture. All measurements were performed in triplicate.

### 2.7 Surface Tension and Critical Micellar Concentration

The Du Noüy ring method was used to measure surface tension at 303.15 K, employing a Krüss EasyDyne K-20 digital tensiometer equipped with a thermostatic bath (Julabo F25-ED). Solutions with varying concentrations of the studied substances were prepared in deionized water, and the equipment was programmed for sequential measurements. Surface tension values were obtained from the mean of 15 consecutive measurements, with uncertainties of 0.01 mN/m for surface tension and 273.25 K for temperature.

The accuracy of the tensiometer was verified by measuring the surface tension of toluene at 293.15 K, yielding a value of 30 mN/m, consistent with literature data. The critical micellar concentration (CMC) was determined from surface tension values at different solution concentrations. The CMC corresponded to the intersection point between two linear regressions fitted to pre-CMC and post-CMC data.^23^

### 2.8 Motor Oil Removal from Sand: Kinetic Testing

The motor oil removal from sand test was conducted exclusively for choline laurate ([CHL]­[LAU]), which exhibited superior surfactant properties. The procedure was adapted from Chaprão et al.^35^

Initially, 10 g of sand were contaminated with 1 g of motor oil in a 50 mL Falcon tube. Then, 20 mL of an aqueous [CHL]­[LAU] solution were added at concentrations of 1/2, 1, and 3 times the CMC. Sodium dodecyl sulfate (SDS) and deionized water were used as positive and negative controls, respectively. The analysis was performed in duplicate.

The tubes were horizontally agitated in a Dubnoff QUIMIS Q226 M bath at level 5 of agitation (150 cycles/min) at 303.15 K for 24 h. After agitation, the supernatant containing the removed oil from the sand was collected, while the remaining sand with residual oil was dried in an oven at 333.15 K for 48 h. The amount of oil removed was determined gravimetrically by comparing the final sand mass to its initial mass before contamination.

### 2.9 Oil Displacement Area

The oil displacement test was adapted from the methodology described by Pele et al.^85^ A Petri dish (9 cm in diameter) was filled with 40 mL of deionized water, followed by the addition of 1 mL of medium crude oil (24° API) on the water surface. The system temperature was raised to 343.15 K on a heating plate to allow the oil to spread uniformly over the Petri dish.

Subsequently, 0.5 mL of a 100 mM aqueous [CHL]­[LAU] solution was added at the center of the oil layer. Sodium dodecyl sulfate (SDS) and deionized water were used as positive and negative controls, respectively. Each test was performed in triplicate, and the diameters of the clear zones were measured using a ruler. Statistical analysis of the data was performed using Microsoft Excel. Mean values were used to calculate the oil displacement area (ODA), following eq 1, as described by Morikawa et al.:^36^

1 ODA=π×(radius)²

### 2.10 Low-Energy Dispersion Test

The low-energy dispersion test was performed with adaptations from the method described by Fernandes et al.^84^ First, 100 mL of aqueous solutions of choline laurate ([CHL]­[LAU]) at concentrations of 100 mmol/L, 50 mmol/L, and 25 mmol/L were placed in 250 mL Erlenmeyer flasks. These concentrations were selected based on the critical micellar concentration (CMC) of [CHL]­[LAU], experimentally determined as 29.9 mmol/L in this study, to evaluate the surfactant’s performance below, at, and above the CMC, and to investigate how micelle formation affects oil dispersion behavior under mild agitation conditions. Then, 100 μL of medium crude oil (24° API) was carefully added to the surface of each solution. The flasks were incubated in a Tecnal TE-424 orbital shaker at 303.15 K and 250 rpm for 24 h.

After this period, an aliquot of the dispersed phase was collected and analyzed using a HAIZ Digital Microscope (50×–1600×) with the HiView software (version 1.4).

### 2.11 Acute Toxicity of Choline Laurate against the Microcrustacean *Artemia salina*

Based on the emulsification index and surface tension results, choline laurate ionic liquid was selected for the acute toxicity test. The procedure was performed according to Miguel et al.^37^

A total of 0.2 g of *Artemia salina* cysts were incubated in 200 mL of a 3.5% w/v NaCl solution (pH 8), under continuous aeration and lighting at 297.15 K, for 48 h. The hatched nauplii were separated by positive phototaxis and transferred to 24-well plates, each containing a final volume of 2 mL.

The nauplii were exposed to five concentrations of the ionic liquid (10, 100, 250, 500, and 1000 μg/mL). The positive control consisted of an aqueous 3.5% w/v NaCl solution, while the negative control used a 0.5 M potassium dichromate solution, selected due to its well-documented and reproducible toxic effects on aquatic organisms, including *Artemia* nauplii. This compound is widely employed as a standard reference toxicant in ecotoxicological studies, providing a reliable benchmark to validate the sensitivity of the assay, as supported by established protocols in the literature by Miguel et al.^37^

Each well contained 10 nauplii, and all treatments were performed in triplicate. After 24 and 48 h of incubation under constant lighting, the number of dead nauplii was recorded. The test was considered valid only if the survival rate in the positive control group was ≥90%.^31^ The mortality percentage was determined and converted into probit values using Microsoft Excel to calculate the lethal concentration (LC_50_) for 48 h of exposure.^38^

## 3 Results and Discussion

After the synthesis of the ionic liquids proposed in this study, their physicochemical characteristics were investigated using Fourier transform infrared spectroscopy (FTIR), as well as density and viscosity analyses. Additionally, their surfactant properties were evaluated by determining the emulsification index and surface tension. The ionic liquid that exhibited the best performance in these properties was further tested for oil spill remediation, assessing hydrocarbon removal from sand and determining the oil displacement area (ODA). Moreover, its acute toxicity against the microcrustacean *Artemia salina* was analyzed.

### 3.1 FTIR Analysis

FTIR spectroscopy provides valuable insights into molecular structures, as each chemical bond exhibits a characteristic absorption wavelength, functioning as a molecular fingerprint.^39^ Figure 1 presents the FTIR spectra of the ionic liquids BTB, 2-HEAP, 2-HEAA, and [CHL]­[LAU] within the range of 650 to 4000 cm^–1^.

**Figure 1:**
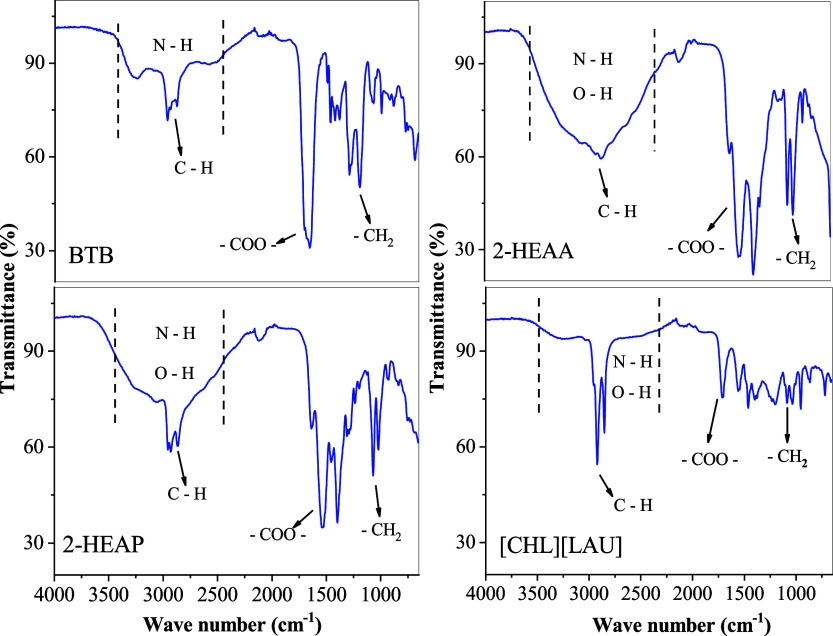
FTIR spectra of ionic liquids [CHL]­[LAU], BTB, 2-HEAP, and 2-HEAA.

A broad band between 3500 and 2400 cm^–1^ is characteristic of the N–H bond and also incorporates the O–H stretching vibration in 2-HEAP, 2-HEAA, and [CHL]­[LAU]. For all the studied ionic liquids, peaks corresponding to the C–H stretching vibrations are observed in the 2800–3000 cm^–1^ region. The presence of asymmetric vibrational modes associated with the carboxylate group (−COO^–^) and the N–H bond is confirmed at 1651 cm^–1^ (BTB), 1535 cm^–1^ (2-HEAA), 1536 cm^–1^ (2-HEAP), and 1707 cm^–1^ ([CHL]­[LAU]). Additionally, characteristic vibrations of the −(CH_2_) group appear at 1289 cm^–1^ and 1192 cm^–1^ (BTB), 1070 cm^–1^ and 1014 cm^–1^ (2-HEAA), 1070 cm^–1^ and 1021 cm^–1^ (2-HEAP), and 1084 cm^–1^ and 1036 cm^–1^ ([CHL]­[LAU]).

These results confirm that the functional groups identified in the FTIR spectra of the synthesized ionic liquids are consistent with their expected chemical structures.

### 3.2 NMR Spectroscopy

The synthesized ionic liquids were characterized by ^1^H NMR spectroscopy. Table 2 presents the chemical shifts of ionic liquids and their simple precursor mixtures, while the corresponding spectra are shown in Figures S1–S8.

**Table 2:** ^1^H NMR Chemical Shifts of the Simple Mixture of ILs Precursors and Produced ILs^a^

	**δ** ^**1**^ **H (ppm)**							
	**BTB**		**2-HEAA**		**2-HEAP**		**[CHL][LAU]**	
#H	**SM**	**IL**	**SM**	**IL**	**SM**	**IL**	**SM**	**IL**
1	7.48	7.46	4.01	4.14	4.26	3.93	0.85	0.86
2	3.39	3.39	3.45	3.48	3.51	3.35	1.22	1.25
3	3.19	3.19	2.68	2.71	2.76	2.70	1.56	1.56
4	2.12	2.12	1.67	1.69	2.06	2.00	2.22	2.23
5	2.23	2.23	-	-	0.92	0.94	3.36	3.28
6	1.49	1.49	-	-	-	-	3.70	3.59
7	1.98	1.98	-	-	-	-	4.11	4.09

a Legend: SM: simple mixture; IL: ionic liquid.

For BTB, a change in the chemical shift was observed only in the singlet of the NH_2_ group. The unchanged chemical shifts of BTB in relation to the simple mixture may be associated with the weak interaction of the precursor ionic groups. However, it may also indicate that the ionic liquid formation reactions occurred rapidly.^32^

In contrast, for 2-HEAA, 2-HEAP, and [CHL]­[LAU], changes in chemical shifts were observed for almost all groups, suggesting the occurrence of chemical interactions. For 2-HEAA and 2-HEAP, for example, the singlet bonded to the nitrogen of the cation shifted from 4.01 to 4.14 ppm and from 4.26 to 3.93 ppm, respectively. These shifts likely result from noncovalent interactions between the amine group of monoethanolamine and the oxygen of the carboxylic acid.^40^

Thus, the results confirm that the synthesized ionic liquids are indeed products of the neutralization reaction rather than mere physical mixtures of their precursors.

### 3.3 Density and Viscosity

Table S1 presents the experimental data of density (ρ) and viscosity (η) of the ionic liquids BTB, 2-HEAA, 2-HEAP, and [CHL]­[LAU] as a function of temperature (298.15–333.15 K) at atmospheric pressure (101.325 kPa). Based on these data and Figures 2 and 3, it is observed that density and viscosity decrease with the increasing temperature, as expected. According to Krisnangkura et al.,^41^ viscosity depends on the interactive force between molecules. As temperature increases, molecular mobility improves, reducing viscosity.

**Figure 2:**
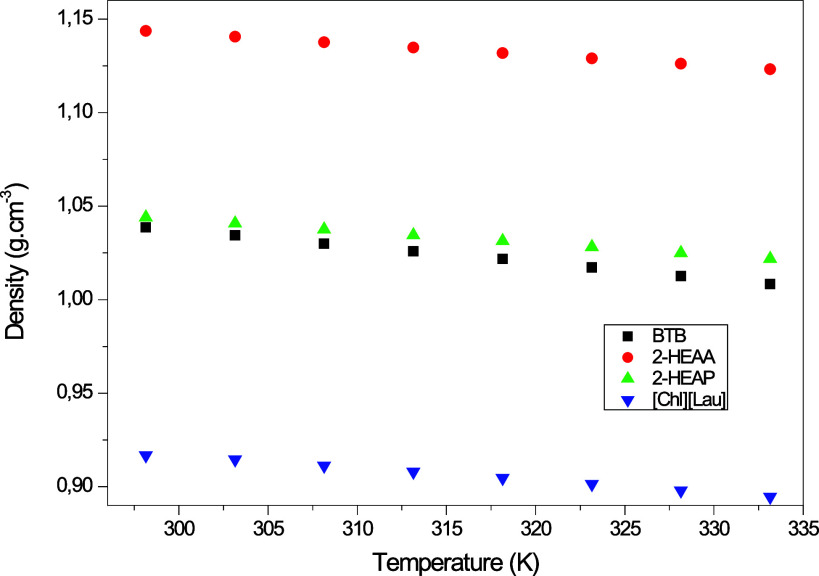
Density (g/cm^3^) of ionic liquids [CHL]­[LAU], BTB, 2-HEAP, and 2-HEAA from 298.15 to 333.15 K.

**Figure 3:**
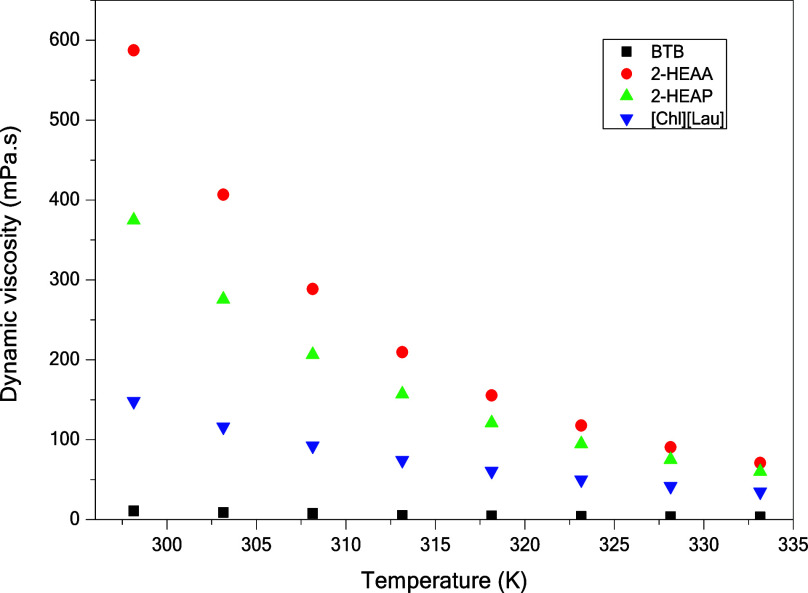
Dynamic viscosity (mPa·s) of ionic liquids [CHL]­[LAU], BTB, 2-HEAP, and 2-HEAA from 298.15 to 333.15 K.

The type of cation and anion in an ionic liquid significantly influences its physical properties. Generally, density decreases as the length of the alkyl chain increases, due to the higher fraction of methylene groups (−CH_2_).^35^ This trend is reflected in the following order: 2-HEAA > 2-HEAP > BTB > [CHL]­[LAU], with choline laurate having the longest alkyl chain and, consequently, the lowest density.

Due to their high molecular weights and strong intermolecular interactions (such as hydrogen bonds, dispersive forces, and electrostatic interactions), most ionic liquids exhibit higher viscosities than common molecular solvents.^41^ For example, 2-HEAA and 2-HEAP ionic liquids showed high viscosity at 298.15 K (587.41 mPa·s and 374.77 mPa·s, respectively). Both contain three hydrogen atoms bonded to nitrogen in their cationic chain, which facilitates cross-linking and increases viscosity.^42^

In contrast, BTB exhibited the lowest viscosity, as lactam-based ionic liquids tend to have weaker ionic interactions due to their lower ability to form strong hydrogen bonds between the nitrogen atoms of the cation and the anion.^29^ This demonstrates that ionic liquids with different viscosities can be tailored by selecting appropriate cations and anions.

From an engineering perspective, viscosity plays a crucial role in mass transfer calculations, modeling, fluid flow, and equipment design, making it a key factor in scaling up applications involving ionic liquids.^43,44^ According to Zhang et al.,^83^ high viscosity is often considered a drawback in laboratory and industrial settings, as it complicates mixing, agitation, separation, and transport operations. Therefore, in the context of oil spill remediation, low-viscosity ionic liquids are preferable, as they can interact more efficiently with the oil and the contaminated surfaces.

### 3.4 Emulsification Index and Emulsion Stability

Emulsions are a dispersion of a liquid in another immiscible liquid, forming small droplets that are typically stabilized by emulsifiers.^46^ In oil spill remediation, emulsion stability is a crucial factor, as dispersed oil droplets must remain suspended to facilitate biodegradation.

Figure 4 illustrates the stability of emulsions formed between the studied ionic liquids and hydrocarbon sources. Commercial surfactant, sodium dodecyl sulfate (SDS), was used as a positive control and deionized water as a negative control.

**Figure 4:**
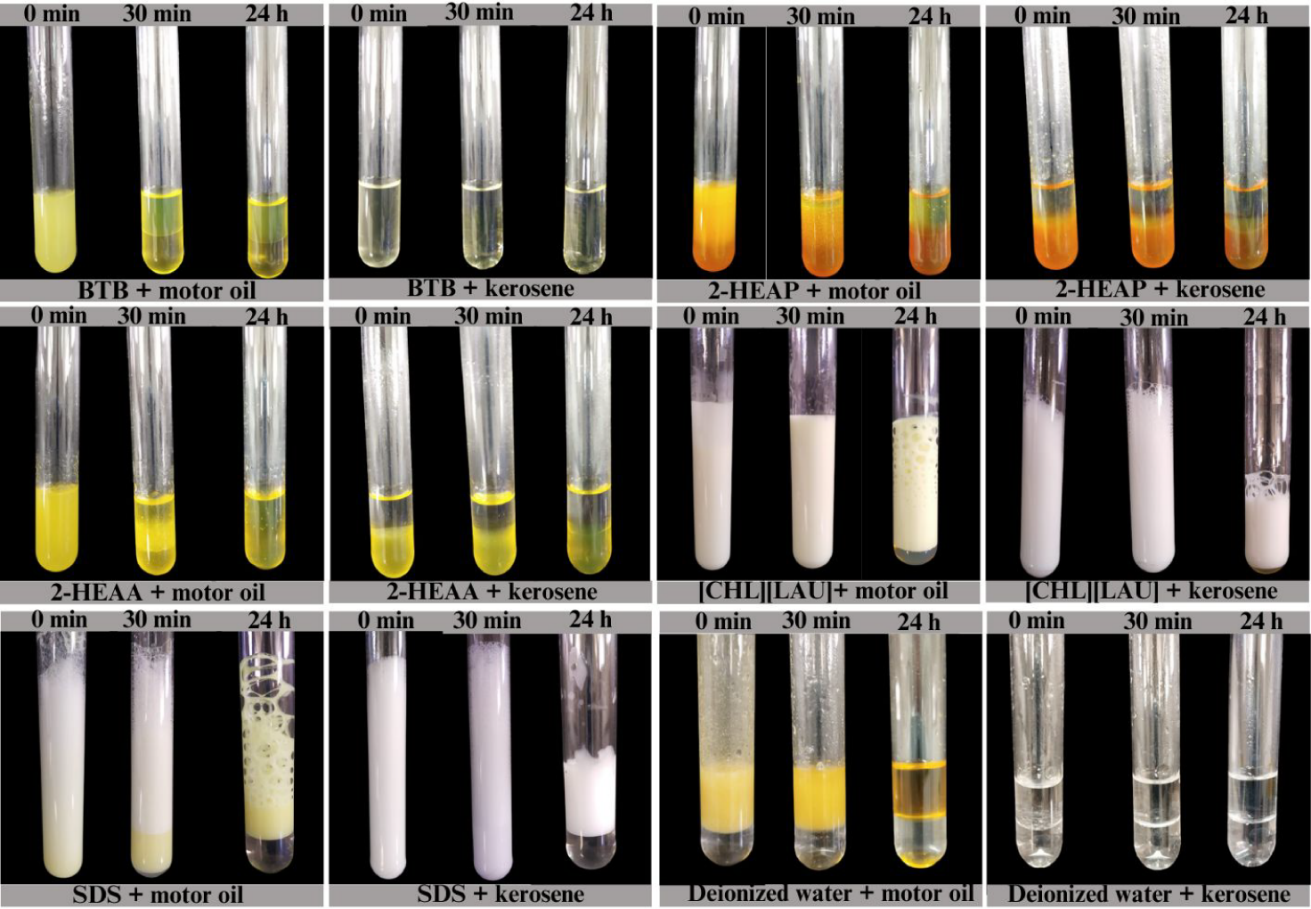
Stability of the emulsion formed between the ionic liquids and the hydrophobic source (motor oil and kerosene).

It was observed that after 30 min, the emulsion formed between BTB and motor oil completely separated into distinct phases, indicating low stability. For kerosene, no emulsion was formed, even under vigorous agitation—this behavior was also observed for 2-HEAA and 2-HEAP. In contrast, with motor oil, both 2-HEAA and 2-HEAP formed an emulsified phase that persisted at the interface for 30 min but fully dissolved after 24 h of rest. These results indicate that BTB, 2-HEAA, and 2-HEAP do not form stable emulsions with the tested hydrocarbon sources.

On the other hand, [CHL]­[LAU] formed stable emulsions with both hydrocarbon sources, allowing for the calculation of the emulsification index (Figure 5). According to Sousa et al.,^47^ an emulsion is considered stable if at least 50% of its original volume remains after 24 h. The emulsions formed by choline laurate remained stable after this period. Additionally, the emulsification index for [CHL]­[LAU] was higher than that of the commercial surfactant SDS for both kerosene and motor oil, highlighting its superior emulsification potential.

**Figure 5:**
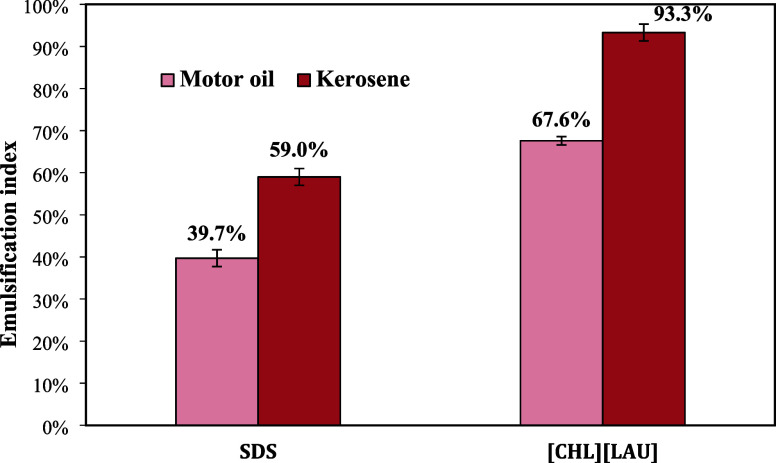
Emulsification index (24 h) of choline laurate ([CHL]­[LAU]) and commercial surfactant SDS against motor oil and kerosene.

Furthermore, the emulsification performance of [CHL]­[LAU] surpassed that of some biosurfactants, which are also being investigated for oil spill remediation. Abdel-Mawgoud et al.,^48^ for example, found an emulsification index (24 h) of 62.5% between the biosurfactant surfactin produced by *Bacillus subtilis* and kerosene. Shahaliyan et al.^49^ found emulsification rates (24 h) of 46% and 6.5% in relation to kerosene, using a mineral salt medium of *Pseudomonas* sp. and *Bacillus* sp., respectively. Nogueira Felix et al.^50^ determined the emulsification index (24 h) between kerosene and surfactin produced by *Bacillus* LAMI002 and obtained a value of 60%. Selva Filho et al.^51^ determined the emulsification index between motor oil and a vegetable biosurfactant produced from *Eichhornia crassipes* extract and obtained a value of 67.4%, close to that of [CHL]­[LAU] (67.6%).

These findings confirm that choline laurate exhibits excellent emulsification properties, making it a promising candidate for application in oil spill remediation.

It is important to note that emulsion stability is influenced not only by the emulsifying potential of surfactants but also by the type of hydrocarbon source. In this study, the emulsification index was higher for kerosene than for motor oil, suggesting that [CHL]­[LAU] forms more stable emulsions with short-chain hydrocarbons. This is likely due to the fact that kerosene consists of lower molecular weight hydrocarbons, with carbon chains ranging from C_8_ to C_16_, whereas motor oil contains a broader range of aliphatic and aromatic hydrocarbons, with chain lengths between C_16_ to C_32_.^52^ This implies that the stability of emulsions formed with crude oil will also be lower, as shown in the study by Ui et al.,^53^ as it is a much more complex and diversified mixture of hydrocarbons.

### 3.5 Surface Tension and Critical Micellar Concentration

The surface tensions of aqueous solutions of the ionic liquids BTB, 2-HEAA and 2-HEAP at 303.15 K were determined by varying their concentrations, as shown in Table 3 and Figure 6. The surface tensions did not follow the expected trend of a continuous reduction with increasing ionic liquid concentration.^54^ This behavior may be due to the absence of ionic liquid molecules on the surface of the solution, as well as their orientation. The reduction in tension is caused by the molecule’s preference to orient itself so that its hydrophilic head faces the interior of the solution, while the hydrophobic tail is directed outward, interrupting hydrogen bonds at the air–water interface.^55,56^ In this regard, the short length of the alkyl chains of ionic liquids butyrolactam butyrate, 2-hydroxyethylammonium acetate, and 2-hydroxyethylammonium propanoate may have influenced this orientation due to lower hydrophobicity.^45^

**Table 3:** Surface Tension (mn/M) of Ionic Liquids BTB, 2-HEAA, and 2-HEAP in Deionized Water at 303.15 K

	Surface tension (mN/m)		
Concentration (ppm)	BTB	2-HEAA	2-HEAP
0	52.0 ± 0.10	52.0 ± 0.10	52.0 ± 0.10
100	46.8 ± 0.07	48.1 ± 0.11	53.3 ± 0.04
200	49.8 ± 0.04	62.0 ± 0.22	56.8 ± 0.06
300	56.6 ± 0.02	60.3 ± 0.05	60.6 ± 0.05
500	50.2 ± 0.04	65.8 ± 0.20	52.5 ± 0.05
800	53.9 ± 0.14	62.4 ± 0.60	51.1 ± 0.00
5000	50.2 ± 0.14	70.8 ± 0.00	59.1 ± 0.20
7000	49.7 ± 0.05	54.7 ± 0.30	51.8 ± 0.00
10000	52.6 ± 0.05	52.6 ± 0.10	52.8 ± 0.04

**Figure 6:**
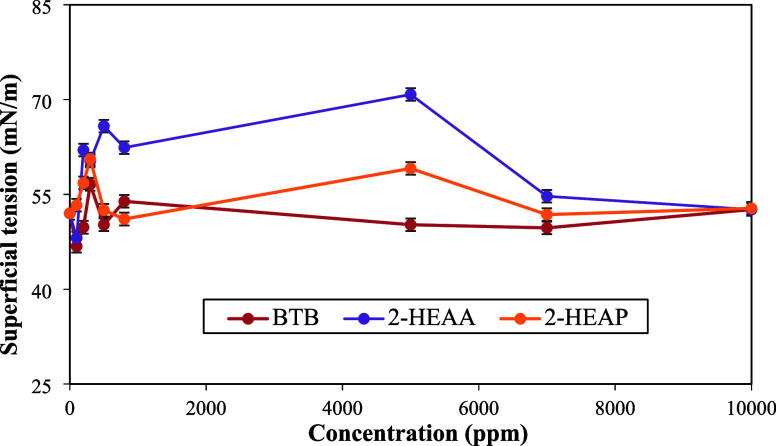
Surface tension of BTB, 2-HEAA, and 2-HEAP in deionized water at 303.15 K at different concentrations.

For BTB and 2-HEAA, there was a small decrease in surface tension at 100 ppm, with minimum values of 46.8 and 48.1 mN/m, respectively, corresponding to reductions of 10% and 7.5% compared to the control (deionized water). For 2-HEAP, the minimum value was 51.1 mN/m at 800 ppm, a reduction of 1.7%. Thus, the maximum reduction in surface tension observed with the ionic liquids in this study was 10%.

Sakthivel et al.^54^ determined the surface tension of an aqueous solution with imidazolium-based aromatic ionic liquids and observed a reduction in tension between 15% and 30%, compared to the control, at temperatures ranging from 288.15 to 318.15 K, showing that they are more efficient than the ionic liquids in this study. This can be explained by the difference between the chain lengths, that is, poor surface tension reduction performances are observed for ionic liquids that have short chains, as is the case of BTB, 2-HEAA, and 2-HEAP.

Surface tension reduction is an important parameter for the effectiveness of a surfactant, as it is related to its ability to adsorb at the air–water interface, minimizing cohesive forces between molecules and influencing surface properties even at low concentrations.^57^ Therefore, it can be concluded that BTB, 2-HEAA, and 2-HEAP do not exhibit strong surfactant capacity, which corroborates the results obtained in the emulsification index.

The surface tension of the choline laurate aqueous solution ([CHL]­[LAU]) was also determined as a function of the ionic liquid concentration, and the result are shown in Figure 7. It is observed that [CHL]­[LAU] reduced the surface tension of water from 52 mN/m to 22.7 ± 0.06 mN/m, corresponding to a reduction of more than 50%. The voltage values were much lower than those obtained for the ionic liquids BTB, 2-HEAP, and 2-HEAA, which may be related to the longer alkyl chain length, favoring stronger adsorption at the air–water interface compared to smaller molecules.^45^ The presence of more methylene groups (−CH2−) in the anionic structure of [CHL]­[LAU] increases its hydrophobicity and surface activity.^58,59^

**Figure 7:**
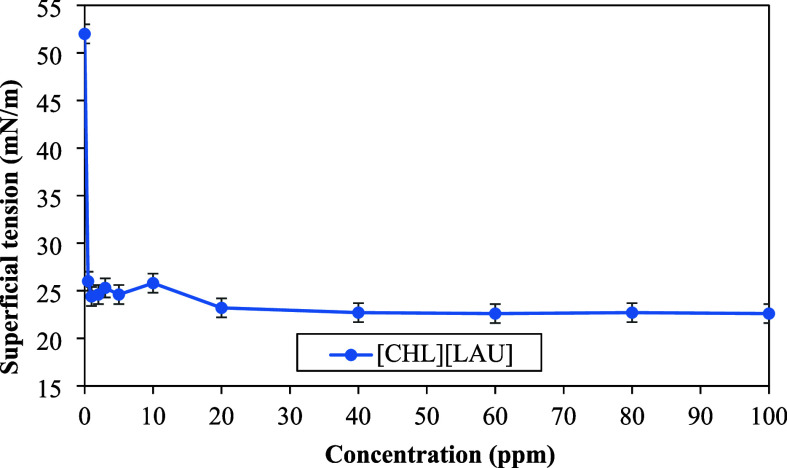
Surface tension of [CHL]­[LAU] in deionized water at 303.15 K at different concentrations.

Compared to data from the literature, [CHL]­[LAU] was also more effective in reducing surface tension than the imidazolium-based surfactant 1-butyl-3-methyl imidazolium chloride, whose minimum tension at 318.15 K was 50.45 mN/m, considering that temperature increase typically decreases surface tension.^54^ Nogueira Felix et al.^50^ reported a minimum tension of 31.8 mN/m using the semipurified biosurfactant produced by *Bacillus subtilis*. Cai et al.^60^ determined the surface tension of aqueous solutions of the chemical surfactant Corexit EC9500A, a chemical surfactant commonly used in oil spill remediation at sea, and obtained a minimum value of 39.96 mN/m at 293.15 K. For sodium dodecyl sulfate (SDS), a surfactant also used in oil remediation, a minimum value of 32.43 mN/m at 308.15 K was found.^61^

In oil spill remediation, surface tension reduction is crucial as it helps disperse oil into small droplets. These smaller droplets have a larger surface area, which facilitates their degradation by native ocean bacteria.^46^

It is evident from Figure 7 that when surface tension reaches a minimum value, significant changes no longer occur, indicating that the critical micellar concentration (CMC) has been reached. It is well-known that important interfacial properties, such as detergency and solubilization, are influenced by the formation of micelles in solution.^62^ The CMC of [CHL]­[LAU] was determined from the intersection found between the lines of the pre- and post-CMC data (Figure S9), obtaining a value of 29.9 mmol/L and a minimum surface tension of 22.7 ± 0.06 mN/m. This result is in close agreement with that obtained by Panda et al.,^63^ who found a CMC of 28.4 mmol/L and a minimum tension of 22.6 mN/m.

It is important to highlight that there is a difference between an effective surfactant and an efficient one. Effectiveness refers to the minimum value to which the surface tension can be reduced, while efficiency is measured by the concentration of surfactant required to significantly reduce the surface tension of water, that is, the CMC.^64^ In this sense, although the CMC of [CHL]­[LAU] is higher than that of conventional surfactants, such as SDS (8.1 mmol/L)^65^ and Corexit EC9500A (1.14 mg/L),^66^ the minimum surface tension achieved is lower, characterizing it as more effective.

### 3.6 Removing Motor Oil from Sand Using Kinetic Testing

Washing using surfactants to remove hydrophobic pollutants that adhere to the surface of soil particles has been proposed as a promising remediation technology, especially because it can be applied as both an ex-situ or in situ process to desorb and concentrate contaminants in the soil without chemically modifying them.^67,68^ In this study, the removal of motor oil from the sand was determined through a kinetic test using aqueous solutions of the surfactant [CHL]­[LAU] at concentrations of 1/2, 1, and 3 times the CMC concentration for washing, respectively. Additionally, sodium dodecyl sulfate (SDS) and water were used as controls. The analyses were performed in duplicate, and the results presented in Figure 8 correspond to the mean and standard deviation of the oil removal percentages obtained for each condition. The error bars included represent this experimental variability, which may be related to the loss of sand particles during the removal of the supernatant.

**Figure 8:**
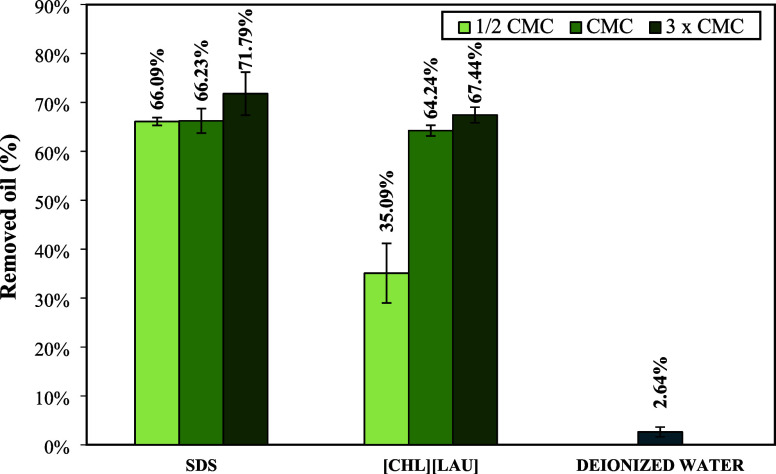
Motor oil removed from sand after kinetic testing by surfactants SDS and [CHL]­[LAU] at 1/2, 1, and 3 times the CMC concentration.

It was observed that increasing the concentration led to more efficient oil removal from the sand, with [CHL]­[LAU] showing a high rate of oil removal at the CMC. SDS removed 66.23% of the oil at the CMC, a result similar to that obtained by Chaprão et al.^35^ 63%, who achieved 63% removal with stirring at 301.15 K and 150 rpm for 24 h.

Oil removal from sand can occur through two primary mechanisms: mobilization and solubilization.^69^ Mobilization works by reducing surface and interfacial tension, thereby decreasing capillary forces and altering the wettability of the solid surface. This makes it easier for oil droplets to detach from the sand. The hydrophobic end of surfactant monomers adsorbs onto the solid surface contaminated with oil, slowly penetrating and diffusing along the oil/solid interface. This forms a hydrophilic adsorption layer, which changes the wettability of the solid surface and facilitates oil transfer to the liquid phase.^70^ This mobilization mechanism occurs at concentrations below the CMC, where the surfactant reduces the surface and interfacial tension between the air/water, oil/water and soil/water systems.

Solubilization, on the other hand, occurs above the CMC, when the solubility of oil increases dramatically due to the aggregation of surfactant micelles. The hydrophobic end of the surfactant molecules clusters within the micelle structure, with the hydrophilic head exposed to the aqueous phase. This enables the hydrophobic interior of the micelle to interact with oil molecules in a process known as solubilization.^67,71^

From Figure 8, it is evident that [CHL]­[LAU] promoted significant oil removal from the sand only at the CMC, indicating that the dominant mechanism is solubilization, driven by micelle formation. In contrast, SDS demonstrated high oil removal both below and above the CMC, indicating that both mobilization and solubilization mechanisms are involved. The high removal efficiency of SDS may be attributed to its chemical structure, as its negatively charged hydrophilic head promotes repulsion between sodium dodecyl sulfate and sand particles. This results in a lower loss of SDS monomers due to soil adsorption compared to the other surfactants tested, whose hydrophilic heads are positively charged.^67^

Figure 9 shows the oil removed from the sand by [CHL]­[LAU], demonstrating that it can be used effectively to remove hydrocarbons from the soil when applied at CMC concentrations.

**Figure 9:**
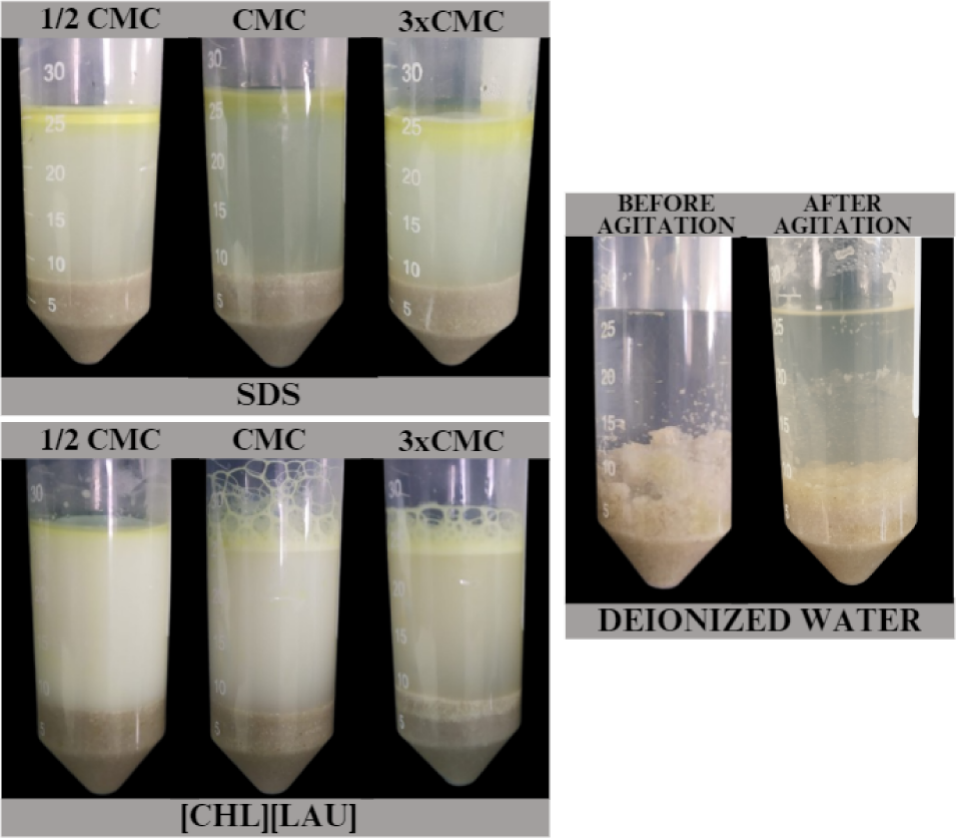
Motor oil removed from the sand after 24 h of agitation (150 cycles/min) at 303.15 K, applying different concentrations of the studied surfactants.

### 3.7 Oil Displacement Area

Oil displacement is a method used to estimate the effectiveness of a surfactant in forming a clear zone during the oil dispersion process.^72^ Figure 10 shows that no dispersion activity was observed when water was added to medium crude oil (24 °API) and motor oil. However, the formation of a halo was observed around both hydrocarbon sources, indicating that the surfactants tested are capable of dispersing the oils.

**Figure 10:**
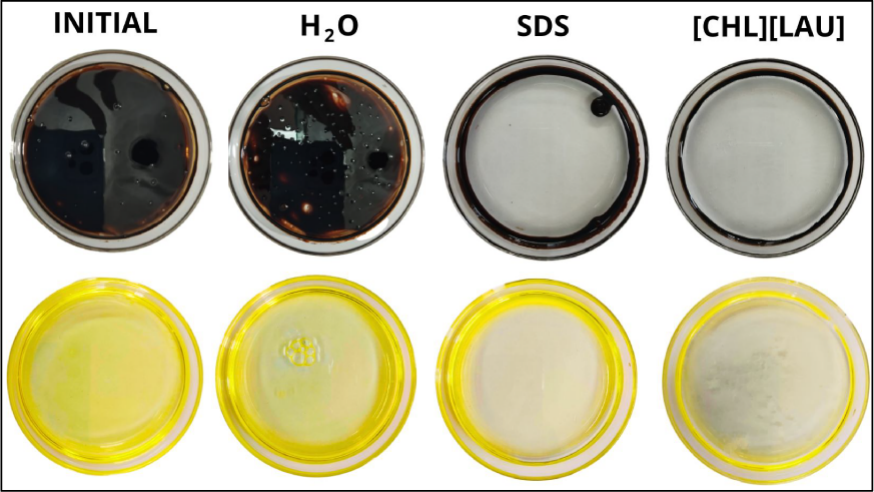
Crude oil and motor oil displacement areas obtained using the surfactants SDS and [CHL]­[LAU] at a concentration of 100 mM.

Considering the total plate area (63.3 cm^2^), choline laurate displaced 93.5% of crude oil and motor oil, while commercial surfactant SDS displaced 75.2% and 73.2%, respectively. Rodriguez et al.^73^ determined the crude oil displacement area by applying a biosurfactant produced by *Bacillus atrophaeus* L193 and obtained a clear zone percentage of 14.2%. da Silva et al.^68^ tested the commercial surfactant Tween 80 and a biosurfactant produced by *Starmerella bombicola*, obtaining displacements of 74.7% and 73.3%, respectively, in relation to crude oil. Satapute and Jogaiah^74^ observed that the biosurfactant surfactin produced by *Lysinibacillus* sp. displaced oil by 50%.

These data demonstrate that [CHL]­[LAU] has a greater ability to displace oil, effectively reducing the surface area covered by an oil slick in a spill, when compared to the other surfactants tested.^75,76^

### 3.8 Low Energy Dispersion Test

Low energy dispersion testing was performed to evaluate the ability of the surfactant to break down the oil slick into smaller droplets. The orbital agitation used promotes moderate shear, simulating the gentle action of waves on an oil slick.^68^ Figure 11 shows that the surfactant was able to disperse the oil, with greater efficiency at a concentration of 100 mmol/L, where the droplets were smaller and more numerous. This occurs because, at the higher concentration, the dispersion was better stabilized, preventing the coalescence of the oil droplets. In the context of an oil spill, choline laurate would be able to disperse the oil, transporting it below the water’s surface in the form of droplets, which can be more easily degraded by the microorganisms present in the sea.

**Figure 11:**
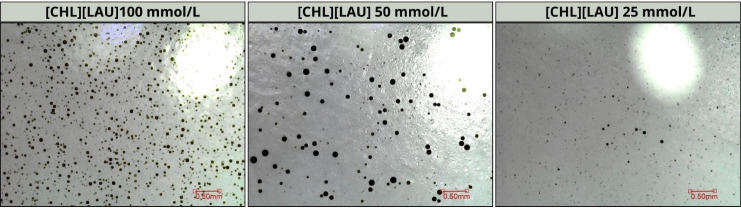
Crude oil dispersions in aqueous choline laurate solutions at 100 mmol/L, 50 mmol/L and 25 mmol/L using the low energy dispersion test.

### 3.9 Acute Toxicity of Choline Laurate against the Microcrustacean *Artemia salina*

Due to its superior results in emulsification index and surface tension reduction, the ionic liquid choline laurate was selected for the acute toxicity test against the microcrustacean *Artemia salina*. This invertebrate is commonly used as a model organism to assess the toxicity of chemicals and natural products in laboratory bioassays, typically by calculating the mean lethal concentration (LC50). Additionally, this test is easy to perform, fast, and low-cost,^77^ and has previously been employed to determine the toxicity of ionic liquids.^78–80^

Figure 12 shows that after 24 h of exposure, no toxic effects of the choline laurate ionic liquid were observed in *Artemia salina* nauplii at concentrations of 10, 100, 250, and 500 μg/mL. After 48 h, dead individuals were found at all concentrations tested, but lethality remained below 25% at concentrations ranging from 10 to 250 μg/mL. At the highest concentration (1000 μg/mL), 56.6% and 73.3% of the nauplii died after 24 and 48 h of exposure, respectively. The positive control (0.5 M potassium dichromate) caused 100% lethality within the first 24 h, while the negative control showed no mortality at any time.

**Figure 12:**
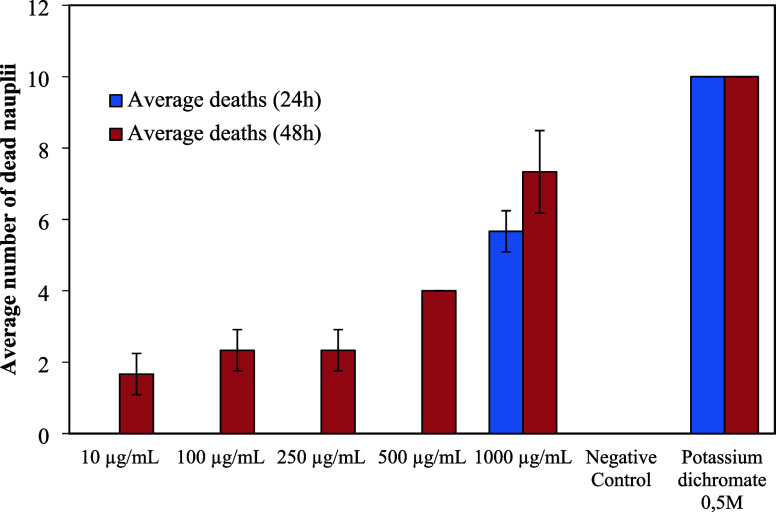
Number of deaths of *Artemia salina* nauplii as a function of the concentration of the ionic liquid choline laurate at 24 and 48 h.

To accurately assess the toxic effect of the ionic liquid, the LC_50_ was determined (Figure S10). The concentration of choline laurate that killed 50% of the subjects within 48 h was 763 μg/mL. According to Directive 93/67/CEE of the European Union,^19^ which classifies substances based on their LC50, the ionic liquid studied is considered nontoxic (LC50 > 100 μg/mL). This result stands in contrast to some commercial surfactants widely used as detergents and emulsifiers, such as sodium dodecyl sulfate (SDS), sodium dodecylbenzene sulfonate, sodium alkyltrioxyethylene sulfate, decaoxyethylene alkyl ether, and nonylphenylheptaoxyethylene glycol ether, which exhibited the following LC50 values against *Artemia salina*: 41.04 μg/mL, 40.4 μg/mL, 11.97 μg/mL, 0.62 μg/mL, and 4.54 μg/mL, respectively, demonstrating high toxicity.^81^

The results are consistent with a study by Ul Hassan Shah et al.,^82^ which classified the binary mixtures of choline-based ionic surfactants, choline myristate ([Cho]­[Mys]) and choline oleate ([Cho]­[Ol]), as nontoxic. The LC50 for these mixtures was found to be 500 μg/mL against zebrafish, suggesting that choline-based ionic liquids tend to have lower toxicity.

## 4 Conclusions

The results of this study demonstrated that, among the four synthesized protic ionic liquids (PILs)—BTB, 2-HEAA, 2-HEAP, and [CHL]­[LAU]—only [CHL]­[LAU] exhibited significant performance as a surfactant. This surfactant reduced the surface tension of water from 52.0 to 22.7 ± 0.06 mN/m, representing a reduction of more than 50%, and exhibited a critical micelle concentration (CMC) of 29.9 mmol/L, indicating that a low concentration is sufficient to significantly reduce surface tension. The emulsification index (24 h) against kerosene and motor oil was 93.3% and 67.6%, respectively—both higher than that of SDS (59.0% and 39.7%)—revealing that the emulsions formed were stable. [CHL]­[LAU] was also capable of removing 67.4% of motor oil from sand and displacing 93.3% of motor oil and crude oil slicks, outperforming SDS. In the acute toxicity test using *Artemia salina*, the lethal concentration (LC_50_–48h) was 763 μg/mL, classifying [CHL]­[LAU] as “non-toxic” according to Directive 93/67/EEC (LC_50_ > 100 μg/mL).^19^ Therefore, the data confirm that [CHL]­[LAU] combines surfactant efficiency with low toxicity, establishing itself as a promising and sustainable candidate for application in oil spill remediation. Further studies are necessary, including tests in real and simulated environments with variations in temperature, pH, and salinity; biodegradability assessments; structural optimization of ionic liquids; and cost analysis.

## Supplementary Material



## Notes

Additional experimental data on the density (ρ) and viscosity (η) of pure ionic liquids, ^1^H NMR spectra for all ionic liquids and their physical mixtures, critical micelle concentration (CMC) data for choline laurate ([CHL]­[LAU]), and LC50 data to assess the toxic effect of the ionic liquid (PDF)
