# Supplementary material for: Protic Ionic Liquids as Eco-Friendly Surfactants for Oil Spill Remediation: Synthesis, Characterization, and Performance Evaluation
Source: ACS Omega. 2025 Jun 16;10(25):27069–81. doi: 10.1021/acsomega.5c02170 (PMC12223878; doi:10.1021/acsomega.5c02170)

Supplementary Materials

Protic Ionic Liquids as Eco-Friendly Surfactants for Oil Spill Remediation: Synthesis, Characterization, and Performance Evaluation

Sádwa F. Ribeiro[1], Hiago S. Braga[1], Rilvia S. Santiago-Aguiar[*,1],

[1]Chemical Engineering Department, Federal University of Ceara, Fortaleza, Ceara, Brazil

***CorrespondingAuthor:** Rílvia Saraiva de Santiago-Aguiar, Chemical Engineering Department, Federal University of Ceará, Pici Campus, Bl. 709, 60440-900, Fortaleza - CE, Brazil; e-mail: rilvia@ufc.br; Tel: +55-85-33669611

List of supplementary materials:

- **Table S1**. Density (ρ) and viscosity (η) data of pure ionic liquids BTB, 2-HEAA, 2-HEAP and [CHL][LAU] from 298.15 to 333.15 K and atmospheric pressure.
- **Table S2**. Properties of crude oil used in this study.

- **Figure S1.** ^{1}H NMR spectrum of ionic liquid 2-HEAA.
- **Figure S2.** ^{1}H NMR spectrum of the physical mixture of the ionic liquid 2-HEAA.
- **Figure S3**. ^{1}H NMR spectrum of the ionic liquid 2-HEAP.
- **Figure S4.** ^{1}H NMR spectrum for physical mixing of the ionic liquid 2-HEAP.
- **Figure S5.** ^{1}H NMR spectrum of BTB ionic liquid.
- **Figure S6**. ^{1}H NMR spectrum of the physical mixture of the BTB ionic liquid.

- **Figure S7**. ^{1}H NMR spectrum of the ionic liquid choline laurate ([CHL][LAU]).
- **Figure S8**. ^{1}H NMR spectrum of the physical mixture of the ionic liquid choline laurate ([CHL][LAU]).
- **Figure S9.** Determination of the critical micelle concentration (CMC) of the Choline laurate ([CHL][LAU]).
- **Figure S10.** Concentration that kills 50% of individuals (LC_{50}) in 48 h. *Artemia salina* submitted to different concentrations of choline laurate. y = 0.6425x + 3.148; R^2 = 0.6149; LC_{50} = 763 μg/mL.

Table S1. Density (ρ) and viscosity (η) data of pure ionic liquids BTB, 2-HEAA, 2-HEAP and [CHL][LAU] from 298.15 to 333.15 K and atmospheric pressure.

**Temperature**	**BTB**		**2-HEAA**		**2-HEAP**		**[CHL][LAU]**	
(K)	ρ (g/cm³)	η (mPa·s)	ρ (g/cm³)	η (mPa·s)	ρ (g/cm³)	η (mPa·s)	ρ (g/cm³)	η (mPa·s)
298.15	1.0386	10.64	1.1436	587.41	1.0440	374.77	0.9168	148.16
303.15	1.0343	8.90	1.1406	406.58	1.0408	275.57	0.9146	116.18
308.15	1.0300	7.56	1.1376	288.47	1.0376	206.22	0.9113	92.31
313.15	1.0258	5.07	1.1347	209.52	1.0345	156.81	0.9080	74.35
318.15	1.0217	4.44	1.1318	155.44	1.0314	121.03	0.9047	60.67
323.15	1.0172	3.92	1.1290	117.58	1.0282	94.77	0.9014	50.11
328.15	1.0126	3.41	1.1261	90.56	1.0250	75.11	0.8980	41.64
333.15	1.0083	3.05	1.1232	70.93	1.0218	60.24	0.8946	34.88

Table S2. Properties of crude oil used in this study.

**Temperature (K)**	**Density ($g.cm^{-3}$)**	**Dynamic viscosity (m.Pa.s)**	**Kinematic viscosity ($mm^2.s^{-1}$)**
293.15	0.9019	1095.9	1215.0
313.15	0.8865	57.064	64.373
333.15	0.8730	24.310	27.847
353.15	0.8597	12.782	14.868

Figure S1. ^{1}H NMR spectrum of ionic liquid 2-HEAA.

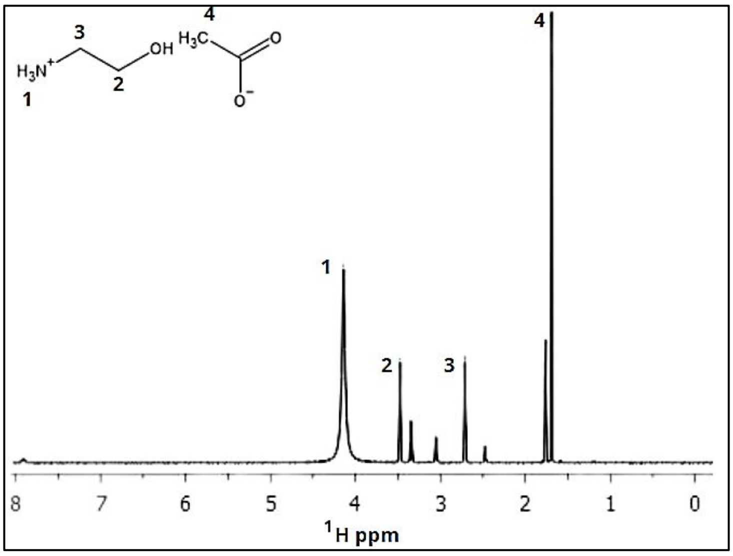

Figure S2. ^{1}H NMR spectrum of the physical mixture of the ionic liquid 2-HEAA.

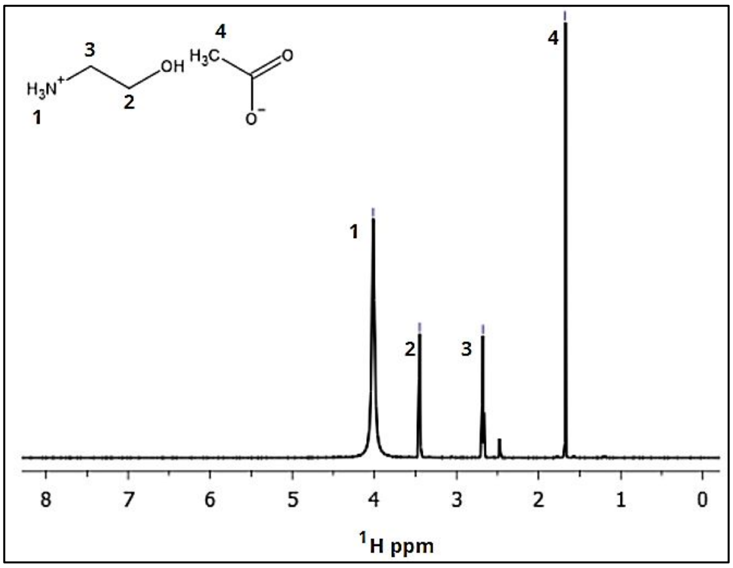

Figure S3. ^{1}H NMR spectrum of the ionic liquid 2-HEAP.

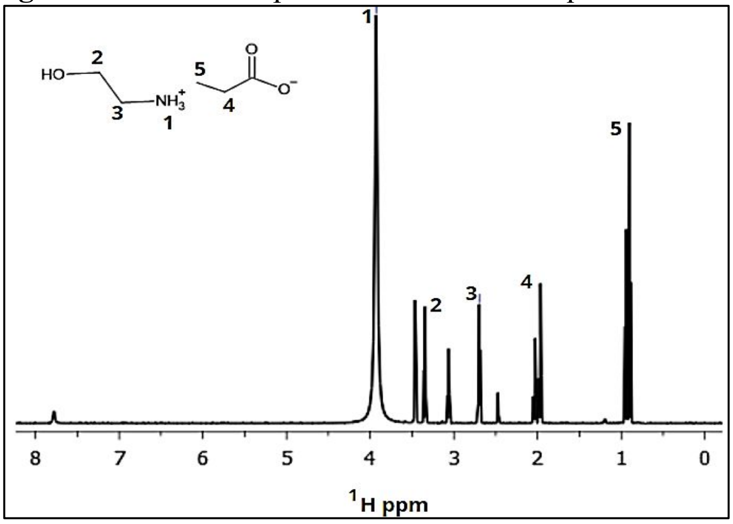

Figure S4. ^{1}H NMR spectrum for physical mixing of the ionic liquid 2-HEAP.

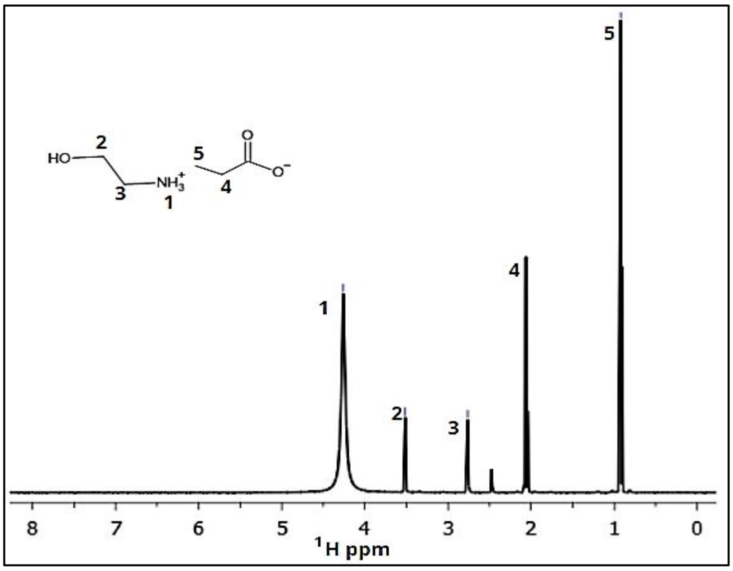

Figure S5. 1H NMR spectrum of BTB ionic liquid

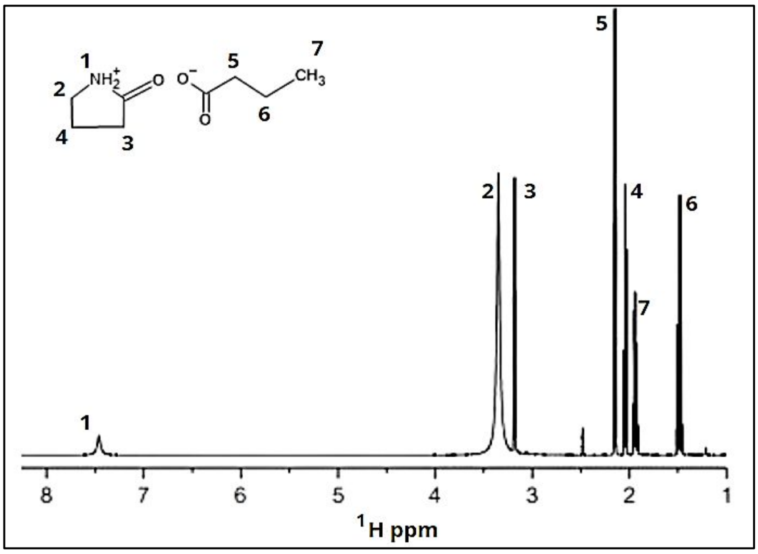

Figure S6. 1H NMR spectrum of the physical mixture of the BTB ionic liquid

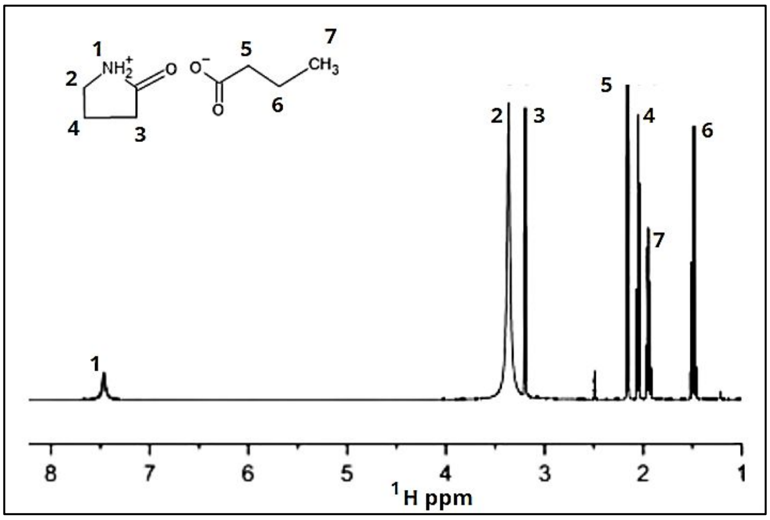

Figure S7. ¹H NMR spectrum of the ionic liquid choline laurate ([CHL][LAU]).

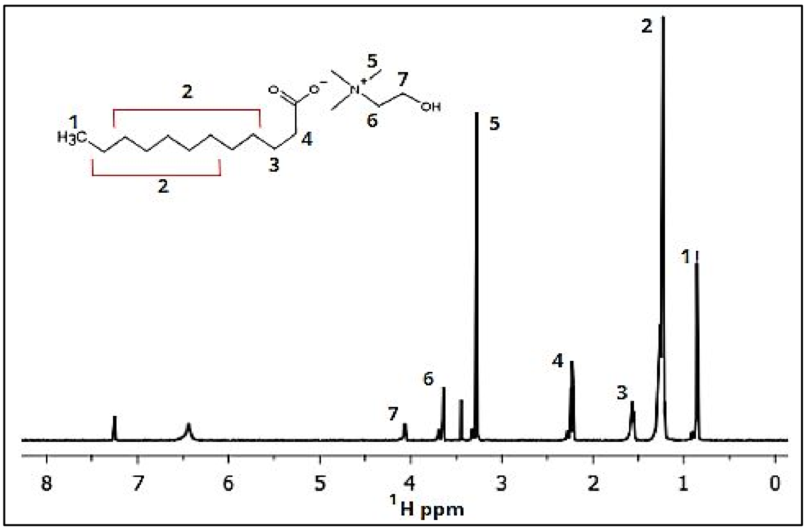

Figure S8. 1H NMR spectrum of the physical mixture of the ionic liquid choline laurate ([CHL][LAU]).

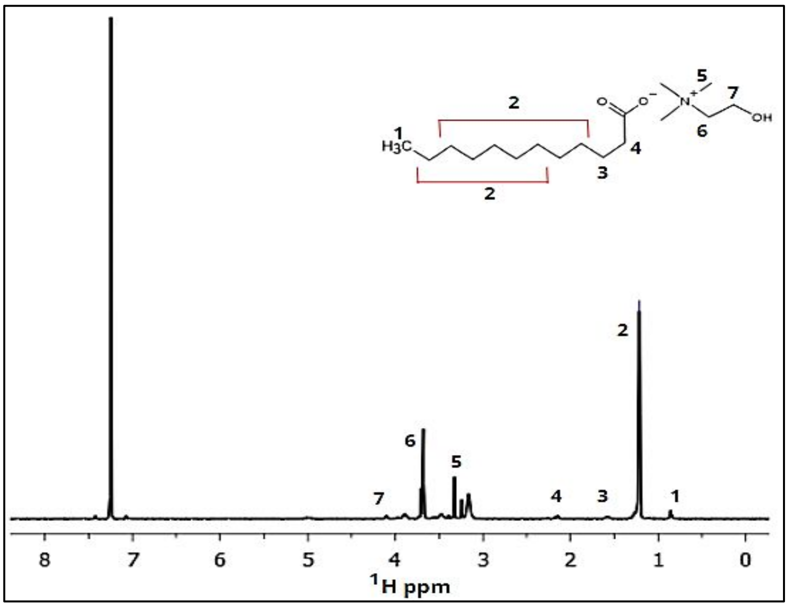

Figure S9. Determination of the critical micelle concentration (CMC) of the Choline laurate ([CHL][LAU]).

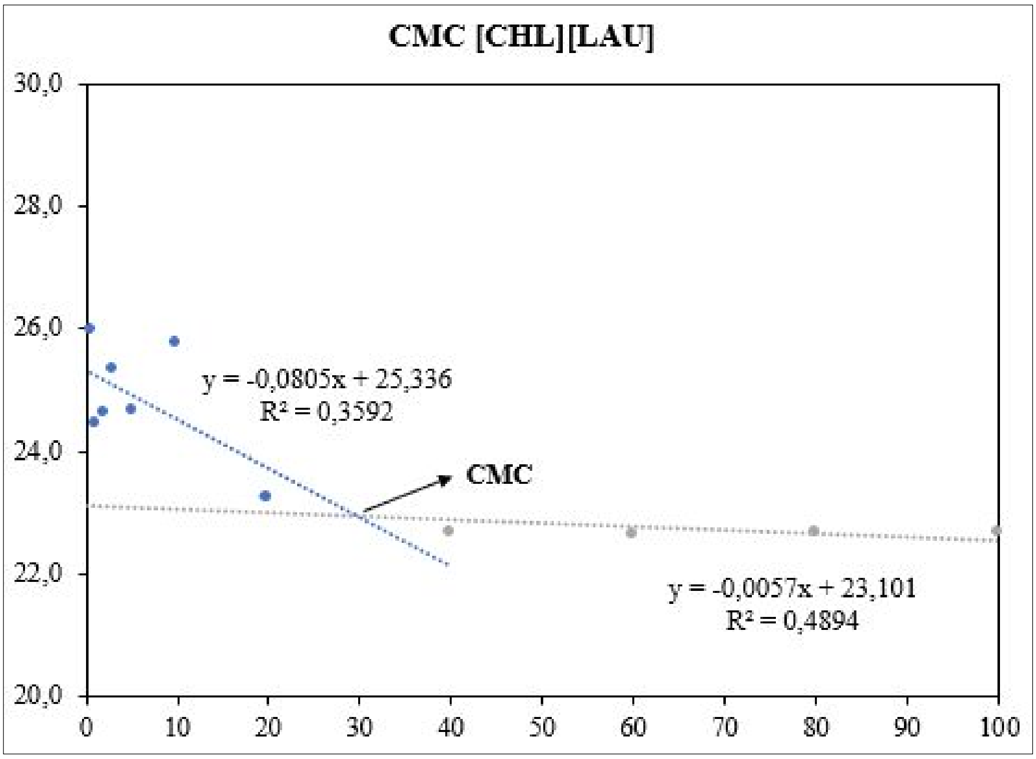

Figure S10. Concentration that kills 50% of individuals (LC_{50}) in 48 h. *Artemia salina* submitted to different concentrations of choline laurate. y = 0.6425x + 3.148; R^2 = 0.6149; LC_{50} = 763 µg/mL.

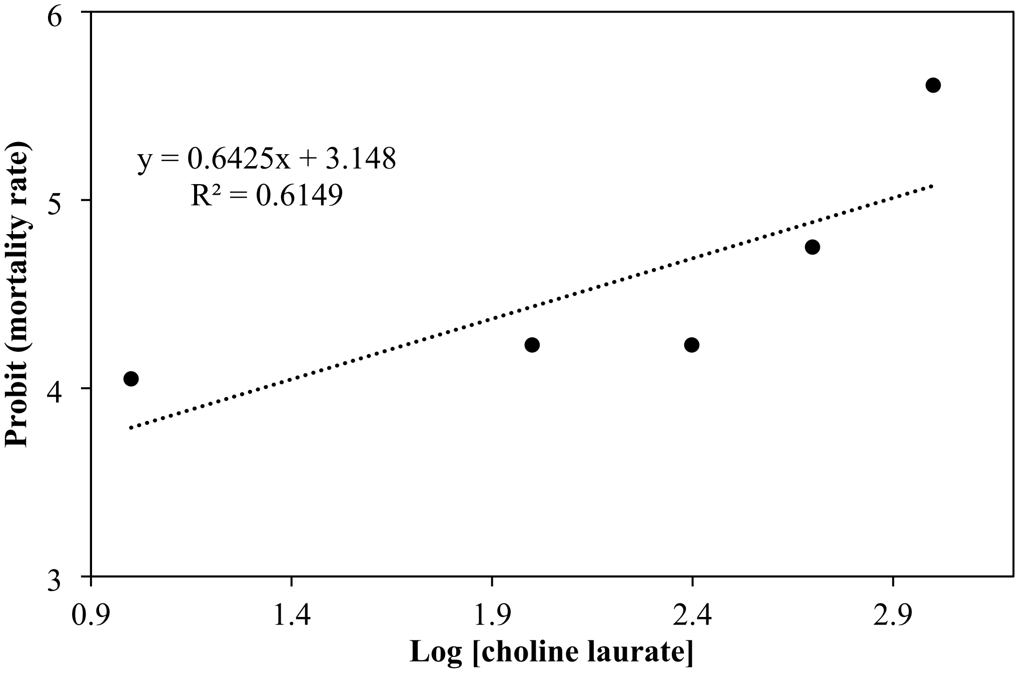

Supplement: Supplementary file 1 [file ao5c02170_si_001.pdf]
